# Surgical Management of Benign Tumors of the Proximal Fibula

**DOI:** 10.5435/JAAOSGlobal-D-21-00207

**Published:** 2021-09-14

**Authors:** Kyle Huntley, Waleed Al-Hardan, Juan Pretell-Mazzini

**Affiliations:** From the Dr. Kiran C. Patel College of Allopathic Medicine, Nova Southeastern University, Fort Lauderdale (Huntley); and the University of Miami Hospital – Jackson Memorial Hospital, Miller School of Medicine, Miami, FL (Dr. Al-Hardan, Dr. Pretell-Mazzini).

## Abstract

Benign tumors of the proximal fibula are clinically notable, often resulting in pain, cosmetic defects, and potential neurovascular compromise. These symptomatic lesions warrant surgical consultation, but specific procedure selection remains a topic of ongoing discussion. The fibula is widely considered an expendable bone, which permits a greater variety of surgical options relative to other skeletal locations. As a result, some authors suggested en bloc resections without reconstruction as a viable first-line option to decrease tumor recurrence risk. However, wide resections may still result in diminished postoperative functionality compared with the standard intralesional and marginal approaches. Thus, surgical management remains a multifactorial decision, and often orthopaedic surgeons rely on past clinical experience or surgical preference within this unique tumor location. This detailed review will summarize the published literature and discuss the outcomes and indications of various surgical approaches for benign tumors of the proximal fibula. Emphasis will be placed on balancing tumor recurrence risk and postoperative functionality within the context of histologic diagnoses and surgical approaches.

Benign tumors of the proximal fibula are clinically notable, potentially causing severe pain, neurovascular compromise, or cosmetic defects that necessitate surgical attention.^1,2,3,4^ Because the fibula is considered an expendable bone, these lesions are afforded multiple, viable management choices compared with benign lesions within other skeletal locations.^1,5-7^ This spectrum of surgical options ranges from minimally invasive procedures, such as intralesional curettage or marginal excision, to more aggressive tissue resections mirroring those of malignant neoplasms. Clinical comparisons of these procedures have been described in the literature with varying outcomes.

Although the proximal fibula contributes minimally to weight-bearing, this anatomic region still presents a surgical risk because of its closeness, proximity to the peroneal nerve, anterior tibial artery, and lateral collateral ligament (LCL) attachment site.^8,9^ As a result, establishing local tumor control and minimizing joint disturbance can pose a challenge for the orthopaedic surgeon.^10^ The ideal surgical outcome maximizes postoperative limb functionality and minimizes the risk of tumor recurrence. Classic thinking has favored minimally invasive excisions over wide resections in the fibula for this purpose, although categorical indications for deciding among procedures have not been established because of a paucity of large-scale comparisons.^11,12^ Accordingly, surgeons frequently rely on clinical experience and preference to steer treatment recommendations.^12^

In this review, we will briefly discuss the clinical significance of benign tumors of the proximal fibula and examine the available options for surgical management within this unique anatomic location. Importantly, we will highlight the risks and postoperative complications surgeons should consider when deciding between specific surgical approaches.

## Epidemiology

In a retrospective review of 9,200 patients diagnosed with a primary skeletal tumor, the fibula was the seventh most frequent site, accounting for 2.6% of bone lesions.^11^ Accordingly, few large-scale epidemiologic studies have thoroughly examined fibula tumor characteristics such as grade, subtype, and anatomic location. To date, one of the most comprehensive analyses was performed by Arikan et al, which included 264 fibular tumors stratified by the Enneking staging system.^13,14^ Of these, most (n = 209; 79.2%) were benign, ranging from stages 1 to 3. The median age of patients with benign tumors was 17 years, with a female predominance of 59%. The proximal third of the fibula was the most common site for benign lesions (67.5%), followed by the diaphysis (24.9%), and the distal third (8.2%). The most frequent benign fibular lesions were osteochondromas, enchondromas, and aneurysmal bone cysts (ABC). In this study, giant cell tumors (GCT) were less commonly reported relative to other smaller scale retrospective analyses (Table 1).

**Table 1:** Studies Reporting the Incidence of Benign Fibular Tumors

Tumor Type	Total Tumors	Arikan et al^13^	Abdel et al^1^	Sun et al^4^	Kundu et al^10^	Dieckmann et al^8^
Osteochondroma	132	51	46	24	8	3
Giant cell tumor	60	6	23	7	22	2
Enchondroma	55	37	11	7	—	—
Aneurysmal bone cyst	54	36	10	1	4	3
Fibroma	36	28	8	—	—	—
Unicameral bone cyst	27	20	7	—	—	—
Fibrous dysplasia	21	15	6	—	—	—
Osteoid osteoma	12	9	2	1	—	—
Intraosseous ganglion	3	1	2	—	—	—
Chondroblastoma	3	—	1	2	—	—
Osteoblastoma	3	—	—	2	—	1
Ollier disease	2	—	2	—	—	—
Intraosseous lipoma	2	2	—	—	—	—
Hemangioma	2	1	—	—	—	1
Maffucci	1	—	1	—	—	—
Eosinophilic granuloma	1	—	1	—	—	—
Nonossifying fibroma	1	—	1	—	—	—
Benign fibrous histiocytoma	1	1	—	—	—	—
Desmoid tumor	1	1	—	—	—	—
Chondromyxoid fibroma	1	1	—	—	—	—
Patient count	418	209	121	44	34	10

## Anatomy

The proximal fibula is surrounded by neurovascular and anatomical structures, which necessitate careful surgical attention.^10^ The peroneal nerve passes over the fibular neck before dividing into the superficial and deep branches, respectively. Damage to the peroneal nerve may result in sensation deficits over the lateral lower leg with a resultant weakness of foot dorsiflexion and eversion.^15^ Similarly, the anterior tibial artery runs adjacent to the proximal fibula and the deep peroneal nerve through the intraosseous membrane.^16^ The proximal fibula contributes to lateral knee stability by serving as the attachment site for the LCL and biceps femoris tendon (BFT). Resection of these tissues off of the proximal fibula can permit varus joint laxity and potentially result in knee instability.^17,18^ Although debated, the role of the proximal fibula in *direct* weight-bearing seems minimal, leading to its general classification as an expendable bone.^12,19,20^ Cadaveric analyses demonstrate that only 7.12% of weight from the knee is transferred through the fibula, which drops to less than 1% when the fibular head is resected.^21^ However, through its contribution to the proximal tibiofibular joint, the fibula dissipates torsional stress at the ankle and alleviates lateral bending of the tibia, and resections of the fibular head may compromise joint stability.^22-24^

## Pathology

Benign skeletal tumors represent a heterogeneous disease spectrum with three general pathologic classifications based on tumor cell type: (1) osteoid-forming, (2) cartilage-forming, and (3) vascular and connective tissue differentiation.^25^ Of the osteoid-forming lesions, osteoid osteomas are the most common variant in the proximal fibula (Table 1). These tumors have a small, cortical nidus that produces haphazard woven bone and is less than 2 cm in diameter.^26^ Although painful, osteoid osteomas do not progress to malignant lesions.^2^ Enchondromas and osteochondromas are the most frequent cartilage-producing tumors of the proximal fibula. Enchondromas consist of lobular hyaline cartilage within the medulla, and care must be given to differentiate their histology from chondrosarcomas.^27^ By contrast, osteochondromas may potentially transform into a secondary chondrosarcoma.^28^

ABCs are common fibular lesions composed of vascular tissue and blood-filled cysts. These tumors are locally destructive but do not undergo malignant transformation.^29^ Some neoplasms such as GCTs of bone do not correspond to a general pathologic classification. GCTs of bone are poorly differentiated benign aggressive tumors characterized by multinucleated osteoclast-like cells. Although they are classified as benign, GCTs of bone may rarely metastasize to the lungs.^30^ By recognizing the unique pathologies of common benign fibula tumors, surgeons can better tailor a management plan for optimal control.

## Clinical Manifestations and Diagnosis

In a review of 120 patients diagnosed with a benign proximal fibula tumor and managed surgically, localized pain (94%) was the most frequent presenting symptom, with a palpable mass (39%), pathologic fracture (17%), or peroneal nerve compression (12%) representing other complaints.^1^ In a cohort of 44 patients, Sun et al^4^ reported that the frequency of a palpable mass (56.8%) and the duration of symptoms (11.7 months) were greater in benign proximal tumors compared with malignant lesions, although statistical significance was not reported. The authors also concluded pain as nonpredicative factor for benign or malignant lesions (*P* = 0.971). Both of the aforementioned studies may be limited by only including patients who were managed surgically; asymptomatic patients were possibly underrepresented.

The extent of benign tumor symptoms typically correlates with their Enneking stage progression; stage 1 tumors are often asymptomatic and incidentally discovered while stage 2 through stage 3 lesions present according to their extent of tissue involvement.^31^ Characteristic manifestations may be seen in select tumors, for example, osteoid osteomas present with nocturnal pain relieved by NSAIDs, and osteochondromas can be associated with limb deformities.^25^

Many benign tumors have characteristic features, and often plain radiographs in multiple planes are sufficient to establish a diagnosis.^32,33^ The nonaggressive benign lesions typically demonstrate a sharp transition zone, a well-defined sclerotic border, and a lack of cortical destruction.^33^ By contrast, various aggressive benign tumors such as GCTs of bones may mimic malignant lesions on plain radiography and require magnetic resonance or CT imaging to aid in diagnosis.^31,34^ If a specific lesion still cannot be identified, a biopsy is generally required to make a definitive histologic diagnosis.^31,34^ Care must be taken to avoid the peroneal nerve.^35^

## Surgical Management

Benign tumors of the proximal fibula can undergo multiple surgical treatments, each with varying risks. The LCL and surrounding neurovascular structures represent surgical considerations when trying to achieve a low recurrence rate and optimal functional outcome.^36^

### Nonsurgical Considerations for Fibular Tumors

The fibula's general classification as an expendable bone has resulted in expanded options for surgical management; however, most asymptomatic benign tumors of the proximal fibula are treated nonsurgically.^13^ Given the paucity of current guidelines or algorithms for this anatomic location, surgical management is largely reserved for certain symptomatic, benign aggressive, and low-grade tumors.^13^ For example, enchondromas represent the most frequent Enneking stage 1 tumor of the proximal fibula, and these often asymptomatic lesions are initially managed with observation and serial radiographs.^25^ Surgical intervention through curettage is classically indicated on the onset of symptoms, increased tumor growth, pathologic fractures, or evidence of a chondrosarcoma.^25^ Similarly, osteoid osteomas have similar clinical outcomes when treated with either NSAIDs or surgical resection, and approximately 50% of tumors spontaneously regress with conservative management.^37,38^ Although excision or radiofrequency ablation of the nidus proves curative and is routinely practiced in other anatomic locations, the increased risk of peroneal nerve involvement makes these invasive procedures less desirable within the proximal fibula.^26,38-40^ Although nonsurgical interventions are a viable initial treatment for select latent lesions, surgical interventions serve the primary role in definitive tumor management in some cases of the proximal fibula.^1,10,41^

### Overview of Surgical Techniques

Four fundamental tumor removal techniques have been described in the proximal fibula: intralesional excision, marginal excision, Malawer type I en bloc resection, and Malawer type II en bloc resection.^1,8,9,41,42^ Each procedure permits limb salvage, and they are differentiated by the extent of tissue resected. Intralesional excisions dissect a portion of the tumor, leaving potentially microscopic neoplastic cells in situ. This technique can be done through curettage, and the tumor cavity is typically filled with bone graft or substitute.^31^ Adjuvant therapies are routinely pursued, particularly for aggressive benign tumors, in an attempt to decrease local recurrence. Cement, such as polymethylmethacrylate, is considered a useful tool to achieve local tumor control through heat generation combined with structural stability.^43-45^ Argon laser, phenol, liquid nitrogen, and other cytotoxic agents are also used and have shown efficacy in decreasing the recurrence rates in the literature.^45,46,47,48^ Marginal excisions cut through the pseudocapsule, potentially leaving microscopic disease, but generally carry less recurrence risk than intralesional procedures.^1,49^

**Figure 1:**
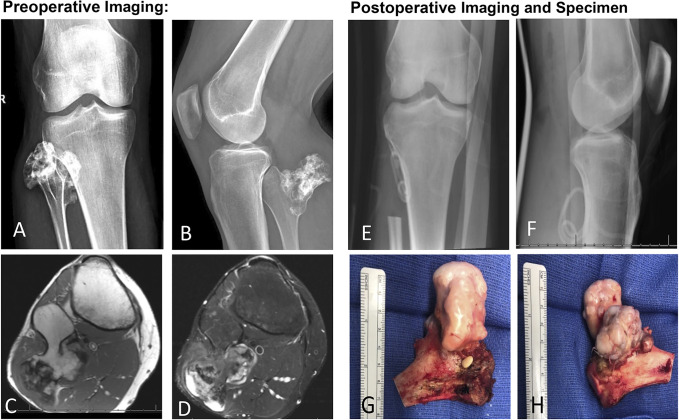
Illustration showing type I resection of osteochondroma. Case presentation: 33-year-old male patient presented with a right knee mass and progressively worsening right foot drop. On imaging, the patient was diagnosed with an osteochondroma. A type I resection was done that resulted in a dramatic improvement of his symptoms. **A, B**, AP and lateral radiographs of the right knee, respectively, showing an osteochondroma involving the proximal fibula with extensive calcifications. **C, D**, Both axial MRI cuts in T1 and T2 sequences showing the cartilage cap of the tumor. **E, F**, Postoperative AP and lateral radiographs showing a type I resection of the proximal 7.5 cm of the fibula with reconstruction of the lateral collateral ligament of the knee. **G, H**, Anterior and posterior views of the gross specimen showing that the fibular medullary cavity is continuous with the medullar portion of the tumor, which is covered superficially by a blue-gray cartilage cap.

Two categories of fibular en bloc resection were originally described by Malawer.^41^ The type I procedure involves a complete resection of the proximal head of the fibula with 2 to 3 cm of healthy proximal diaphysis (Figure 1). A thin layer of musculature, when possible, is circumferentially removed in addition to the LCL and BFT attachment sites. The LCL and BFT can be reanchored to surrounding tissues, typically the tibial metaphysis.^31,41,42^ The peroneal nerve and anterior tibial artery are preserved. By contrast, type II resections sacrifice the peroneal nerve, anterior tibial artery, and 6 cm of proximal healthy diaphysis.^41^ In type II resections, the biceps tendon and LCL are resected 2.5 cm proximal to their fibular attachment site, possibly complicating reattachment.^36,41^ Attempts to revise the Malawer dichotomy have been proposed; Erler et al^9^ and Dieckmann et al^8^ endorsed supplementary resection techniques based on the tumor size and quantity of structures removed, respectively. Despite these proposed refinements, the Malawer criteria remain prevalent as foundational resection procedures.^31^

Generally, type I resections (Figure 1) have been used for benign aggressive tumors of the proximal fibula, such as GCT of bone, while intralesional and marginal excisions are the choice for less aggressive lesions. Type II resections for benign growths are rare but have been described (Table 2).^13^ Despite this pattern, procedure selection remains a multifactorial decision. The surgeon must account for tumor type, size, probability of recurrence, and postoperative functional outcomes (including potential peroneal nerve palsy and ligamentous instability).^7,9,10,12^

**Table 2:** Choice of Surgical Management With Local Recurrence Rates for Common Benign Tumors of the Proximal Fibula; Results Aggregated From Abdel et al,^13^ Sun et al^a^,^4^ Guo et al^a^,^21^ Kundu et al,^10^ and Inatani et al^55^

Tumor Type	Intralesional	Local Recurrence	Marginal	Local Recurrence	Type I	Local Recurrence	Type II	Local Recurrence
Osteochondroma	7	1 (14%)	58	0	25	0	—	0
Giant cell tumor	7	4 (57%)	0	0	35	3 (9%)	14	0
Enchondroma	2	0	2	0	14	0	—	0
Aneurysmal bone cyst	3	3 (100%)	3	0	8	0	—	0
Fibroma	6	0	—	0	1	0	—	0
Unicameral bone cyst	5	0	—	0	1	0	—	0
Total (% recurrence)	30	8 (27%)	63	0 (0%)	84	3 (4%)	14	0 (0%)

a Analyses used the same patient sample; data regarding procedure selection (Sun et al^4^) and tumor recurrence (Guo et al^21^) were reported separately.

### Surgical Considerations: Recurrence Rate and Tumor Type

For most benign tumors of the proximal fibula, intralesional and marginal excision procedures have shown similar postoperative tumor recurrence rates as en bloc resections (Table 2).^1,21^ Guo et al^21^ retrospectively analyzed 44 patients with benign proximal fibula tumors who underwent either intralesional excision or a type I resection and identified potential risk factors for local recurrence. One GCT of bone managed by a type I resection was the only recurrence within the benign tumor cohort, and there was no statistical difference in recurrence rate between the intralesional and type I treatment groups. When the investigation was expanded to include eight additional patients with *malignant* proximal fibular neoplasms, univariate testing demonstrated peroneal nerve palsy on presentation and malignant status as the only variables associated with tumor recurrence (*P* < 0.01). In addition, when the patient variables were independently controlled for in a multivariate analysis, including the method of surgical management and tumor type, peroneal nerve palsy on initial presentation was the only predictive variable for tumor recurrence (*P* < 0.01). Although statistically significant, this study was potentially limited by a small sample size and a lack of stratification between pathologic subtypes.

To date, Abdel et al^1^ conducted the largest study directly comparing recurrence rate by the surgical technique. Their study included 121 benign tumors (120 patients) of the proximal fibula, which were managed by intralesional curettage, marginal excision, or type I resection. Overall, the recurrence rate was 8% (n = 10), with recurrence statistically more frequent in the intralesional curettage group compared with the en bloc group (23% versus 5% *P* = 0.029). However, when stratified by tumor pathology, GCTs of bone and ABCs made up most tumor recurrences (70%).

Within the GCT of bone group, patients treated with intralesional excision experienced a higher recurrence rate compared with those managed by an en bloc resection (67% versus 11% *P* = 0.08). In addition, all patients with an ABC who were managed through intralesional curettage experienced tumor recurrence, whereas none of those treated with en bloc resection had recurrence (100% versus 0%; *P* = 0.008). Accordingly, Abdel et al^1^ concluded that although many tumors can be appropriately managed with intralesional excision, GCT and ABC warrant consideration for en bloc resection.

Although there were zero recurrences in the marginal resection group in the study of Abdel et al,^1^ this result can likely be attributed to the selection of tumor pathologies treated, rather than the surgical technique performed. Within the marginal group, osteochondromas were disproportionally the most common tumor treated (28/32; 87.5%) compared with the intralesional group (7/30; 23.3%) and type I resection group (11/56; 19.6%). In general, osteochondromas demonstrate low recurrence rates of less than 2% after surgical intervention.^50,51^ In addition, the marginal excision group did not include GCT of bone nor any cases of ABC, both benign aggressive tumors with a higher risk of recurrence. Thus, it is likely that the recurrence results within the marginal cohort of this study were aided by a relatively favorable distribution of tumor histology; this remains a notable limitation when attempting to directly compare the efficacy of different surgical techniques.

### Tumor Type Considerations

#### Benign Nonaggressive Tumors of the Proximal Fibula

Benign nonaggressive tumors within the proximal fibula, such as osteochondromas, enchondromas, simple bone cysts, and chondroblastomas, demonstrate a low risk of recurrence after intralesional and marginal excisions (Table 2).^1,21^ Accordingly, if surgery is the desired management choice for stable nonaggressive tumors in this anatomic location, the intralesional approach is a recommended initial surgical intervention.^1,31^ Dahlin et al^52^ reported on 30 cases including chondroblastoma with a cure rate of 90%, with the remaining recurrences adequately treated by revision curettage and no noted surgical complications. Similarly, in a study by Bauer et al,^53^ enchondromas demonstrated a recurrence rate of 4% after intralesional curettage. As discussed previously, adjuvant therapies can be used for the benign nonaggressive tumors in this anatomical region to further control recurrence risk.^45,46,47,48^ Given the low reported recurrence rates, intralesional and marginal excisions provide the added benefit of decreased risk of iatrogenic neurovascular and anatomical compromise in this sensitive region.^10^ En bloc type I resections have been described for benign nonaggressive tumors of the proximal fibula but are typically reserved for rapidly growing, locally destructive lesions with potential for malignant transformation.^7,10,31,54^

#### Benign Locally Aggressive Tumors of the Proximal Fibula

Most authors acknowledged en bloc resection as an effective management option for fibular GCT of bone, whereas others asserted intralesional excision with adjuvant therapy as viable alternatives. Those advocating for en bloc resection of the proximal fibula for GCT's of bone cited both the aggressive nature of the tumor and the high recurrence rates as indications because they outweigh the potential risk of neurovascular or functional complications.^9,10,30,55^ Inatani et al^55^ concluded a 50% recurrence rate after intralesional curettage in four patients with GCT of bone in the proximal fibula. By contrast, smaller case series of patients with fibular GCT of bone managed with intralesional excision reported no recurrences.^56^ In addition, novel tumor curettage techniques in the fibula have resulted in decreased healthy tissue removal while avoiding an increase in recurrence rates of GCT of bone.^48,57^ These techniques use posterior surgical windows and precise endoscopic curettage with cementation and argon plasma coagulation, respectively, and warrant further clinical consideration.^48,57^

Like GCTs of bone, the preferred treatment strategy for ABCs of the proximal fibula remains under discussion. ABCs in nonexpendable anatomical locations, such as the femur, are classically managed with curettage and bone grafting, although recurrence can vary between 10% and 59%.^29,58,59^ Because of the potentially high recurrence rate, some authors cited the expendable nature of the fibula as an indication for en bloc resection. Vergel De Dios et al^60^ examined 238 patients with ABC in varying skeletal locations; there were zero recurrences in those treated with en bloc resection, compared with 19% with intralesional curettage. These findings are agreeable with Abdel et al^1^ and Campanacci et al^61^ who similarly found zero recurrences with en bloc resection. By contrast, small-scale studies and case reports advocate for less invasive strategies. Lampasi et al^62^ and Jesudason et al^3^ concluded zero recurrences of fibular ABC's after intralesional curettage, although sample size was limited to six patients and one patient, respectively. Mavrogenis et al^63^ reported a successful ABC embolization in the proximal fibula with rapid pain relief and no tumor recurrence. Rossi et al^64^ reported on 36 patients and found no recurrence in 97% of patients treated with embolization.

Overall, the available evidence implies limited recurrence risk differences between intralesional/marginal tumor removal and en bloc resections for *most* benign tumors of the proximal fibula, with GCT of bone and ABC being the exception. Although evidence is mixed, larger scale studies support type I en bloc resection of the proximal fibula as the preferred surgical treatment of GCT of bone and ABC, if the goal is to diminish recurrence rates.^1,7,10,29^ However, achieving treatment success remains multifactorial, including surgical skill, use of adjuvant therapies, and extent of the lesion.^7,9,10^

### Functional Outcomes

Although the risk of tumor recurrence remains a key surgical consideration, notable clinical attention should also be given to achieving adequate postoperative mobility, function, and joint stability. Consideration of postoperative functional limitations may influence selection between procedures with similar recurrence rates.

#### Peroneal Nerve

The peroneal nerve plays a major role in postoperative function, and its involvement is associated with inferior outcomes.^8-10,55^ Kundu et al^10^ demonstrated this by using the Musculoskeletal Tumor Society (MSTS) scoring system in their analysis of 46 patients with a proximal fibula tumor treated with an en bloc resection. The authors concluded that those with a concomitant peroneal nerve resection had a significantly lower average MSTS score compared with patients with no nerve involvement (82% versus 93%; *P* < 0.01). Inatani et al^55^ demonstrated an even greater disparity in postoperative function within their cohort because those with a peroneal nerve resection had a mean MSTS score of 65% compared with 96% without involvement (*P* < 0.05), although the analysis may be limited by small sample size (n = 12). Dieckmann et al^8^ and Erler et al^9^ demonstrated analogous results because patients with peroneal nerve involvement resulted in lower functional scores. Even before surgical intervention, patients with evidence of peroneal nerve palsy on initial presentation have a higher risk of wound healing complications including infection.^21^ Thus, it is imperative for the orthopaedic surgeon to weigh the risk of peroneal nerve involvement for each surgical procedure.

The rate of iatrogenic peroneal nerve injury during tumor removal ranges from 3% to 57%.^1^ Although studies providing direct comparisons between procedures remain limited, en bloc resections show a tendency of greater risk of peroneal nerve involvement.^1^ In type I resections, the peroneal nerve is typically protected by mobilizing it from the peroneus longus through sacrifice of the articular branch; nerve traction during surgical retraction is a common source of injury.^10,31^ In the analysis of Abdel et al on 120 patients with benign proximal fibula tumors, 9 (7.5%) reported a postoperative peroneal nerve palsy. The type I resection group resulted in six palsies (n = 56; 10.7%), and the remaining three originated from the intralesional/marginal excision group (n = 62; 4.8%); statistical significance was not reported. Similarly, the authors cited peroneal nerve palsy as a common complication after a type I resection, whereas nerve injury is rarely reported after an intralesional excision.^8,41,42,56,65,66^ Spontaneous peroneal nerve recovery within a year of surgery has been reported because of traction injury.^1,42,66^

#### Lateral Collateral Ligament and Biceps Femoris Tendon

Surgical detachment of the LCL and BFT can provide an additional source of impaired postoperative function, and evidence demonstrates that reattachment after an en bloc resection improves functional outcomes.^31,36,66^

Zhao et al^66^ reported on 19 patients who had a type I resection and compared lateral knee stability and MSTS function scores in patients who either underwent, or did not undergo, subsequent LCL and BFT reattachment to the tibial metaphysis. In their study, the reattachment group was associated with significantly higher rates of lateral knee stability (100% versus 57.1%; *P* < 0.05) and higher scores on MSTS function surveys (97.7% versus 71.8%; *P* < 0.05). Similarly, Bickels and Wittig^31^ reported on 15 patients who underwent a type I resection and tibial LCL reattachment; 14 had complete lateral knee stability with only one patient reporting a grade 1 instability (lateral joint opening of 1 to 5 mm), although no control group was included.

Arikan et al^36^ analyzed six patients who underwent type I resection with LCL and BFT reattachment to the surrounding soft-tissue structures, rather than the tibial metaphysis, and all patients recorded some level of knee instability, varying from grade 1 to grade 2. Agarwal et al^67^ concluded similar rates of instability after LCL reattachment to soft-tissue structures.

Overall, the relative procedural simplicity and lack of adverse events have led to the recommendation that LCL and BFT reattachment should be practiced, preferably through anchoring to the tibial metaphysis, after type I en bloc resections of proximal tibia.^1,31,42,66^

#### Type II Proximal Fibula Resections

Multiple studies have demonstrated that type II resections are associated with markedly higher rates of knee instability and inferior MSTS scores compared with type I resections.^42,66^ This deficiency is largely attributed to the decreased LCL and BFT stump length (9.0 ± 2.5 cm versus 21.7 ± 9.0 cm; *P* = 0.018) and the extent of tissue removed compared with type I resections.^36,42^ An inability to reattach the ligamentous structures further contributes to the instability of the proximal tibiofibular joint, which may also produce secondary deficiencies in ankle mobility.^24^ Accordingly, type II resections are rarely indicated for benign lesions of the proximal fibula, unless extended tumor involvement of adjacent anatomical structures commands a widespread resection.

## Overview

Benign tumors of the proximal fibula are uncommon and can be clinically notable causes of morbidity. The choice of surgical management of these lesions is a multifactorial decision, with recurrence risks, functional outcomes, and patient goals guiding selection.

Intralesional and marginal excision procedures are effective at treating benign nonaggressive tumors of the proximal fibula and are the preferred initial surgical option. These procedures have decreased postoperative functional limitations and no discernible differences in recurrence rates compared with en bloc resections.

By contrast, Malawer type I en bloc resections are the preferred treatment for benign aggressive tumors, such as ABCs and GCTs of bone. Recurrence rates are lower compared with intralesional/marginal excisions, but the invasive nature of this procedure increases the risk of postoperative functional limitations. LCL and BFT reattachment to the tibial metaphysis should be done to improve postoperative joint stability. In patients who desire a more active lifestyle after surgery and find the functional risks unacceptable, intralesional or marginal excisions may be considered an appropriate initial therapy, although recurrence should be closely monitored. All options should be collaboratively discussed with the patient, and the surgical management plan that best balances the oncological and functional outcomes should be selected.

## References

[1] Abdel MP Papagelopoulos PJ Morrey ME Wenger DE Rose PS Sim FH: Surgical management of 121 benign proximal fibula tumors. Clin Orthop Relat Res 2010;468:3056-3062. 20625947 10.1007/s11999-010-1464-8 PMC2947668

[2] Dookie AL Joseph RM: Osteoid osteoma, in the StatPearls. Treasure Island, FL, StatPearls Publishing, 2021.. 30725964

[3] Jesudason P Akhtar S Zenios M: An aneurysmal bone cyst within the proximal fibula causing common peroneal nerve palsy. Musculoskelet Surg 2013;97:173-175. 21822623 10.1007/s12306-011-0161-4

[4] Sun T Wang L Guo C Zhang G Hu W: Symptoms and signs associated with benign and malignant proximal fibular tumors: A clinicopathological analysis of 52 cases. World J Surg Oncol 2017;15:92. 28464896 10.1186/s12957-017-1162-z PMC5414337

[5] Panagopoulos GN Mavrogenis AF Mauffrey C: Intercalary reconstructions after bone tumor resections: A review of treatments. Eur J Orthop Surg Traumatol 2017;27:737-746. 28585185 10.1007/s00590-017-1985-x

[6] Schneiderbauer MM Gullerud R Harmsen WS Scully SP: Fibular osteosarcomas: Contaminated margins may not impact survival. Clin Orthop Relat Res 2007;456:182-187. 16967031 10.1097/01.blo.0000238834.95928.0f

[7] Weisstein JS Goldsby RE O'Donnell RJ: Oncologic approaches to pediatric limb preservation. J Am Acad Orthop Surg 2005;13:544-554. 16330516 10.5435/00124635-200512000-00007

[8] Dieckmann R Gebert C Streitbürger A: Proximal fibula resection in the treatment of bone tumours. Int Orthop 2011;35:1689-1694. 21221575 10.1007/s00264-010-1193-3 PMC3193948

[9] Erler K Demiralp B Ozdemir MT Basbozkurt M: Treatment of proximal fibular tumors with en bloc resection. Knee 2004;11:489-496. 15581770 10.1016/j.knee.2003.10.005

[10] Kundu ZS Tanwar M Rana P Sen R: Fibulectomy for primary proximal fibular bone tumors: A functional and clinical outcome in 46 patients. Indian J Orthop 2018;52:3-9. 29416163 10.4103/ortho.IJOrtho_323_16 PMC5791228

[11] Niu X Xu H Inwards CY: Primary bone tumors: Epidemiologic comparison of 9200 patients treated at Beijing Ji Shui Tan Hospital, Beijing, China, with 10 165 patients at Mayo Clinic, Rochester, Minnesota. Arch Pathol Lab Med 2015;139:1149-1155. 25978765 10.5858/arpa.2014-0432-OA

[12] Perisano C Marzetti E Spinelli MS: Clinical management and surgical treatment of distal fibular tumours: A case series and review of the literature. Int Orthop 2012;36:1907-1913. 22527336 10.1007/s00264-012-1536-3 PMC3427430

[13] Arikan Y Misir A Ozer D: The incidence and distribution of primary fibula tumors and tumor-like lesions: A 35-year experience. J Orthop Surg 2018;26:2309499018798180. 10.1177/2309499018798180 30189775

[14] Enneking WF: A system of staging musculoskeletal neoplasms. Clin Orthop Relat Res 1986;204:9-24. 3456859

[15] Lezak B Massel DH Varacallo M: Peroneal nerve injury, in StatPearls. Treasure Island, FL, StatPearls Publishing, 2021. 31751049

[16] Keith L Moore AFD Anne M. R.Agur: Clinically Oriented Anatomy. ed 8th. Philadelphia, PA: Wolters Kluwer, 2018.

[17] Draganich LF Vahey JW: An in vitro study of anterior cruciate ligament strain induced by quadriceps and hamstrings forces. J Orthop Res 1990;8:57-63. 2293634 10.1002/jor.1100080107

[18] Grood ES Noyes FR Butler DL Suntay WJ: Ligamentous and capsular restraints preventing straight medial and lateral laxity in intact human cadaver knees. J Bone Joint Surg Am 1981;63:1257-1269. 7287796

[19] Bozkurt M Yavuzer G Tönük E Kentel B: Dynamic function of the fibula. Gait analysis evaluation of three different parts of the shank after fibulectomy: Proximal, middle and distal. Arch Orthop Trauma Surg 2005;125:713-720. 16267651 10.1007/s00402-005-0054-9

[20] Lee JH Chung CY Myoung H Kim MJ Yun PY: Gait analysis of donor leg after free fibular flap transfer. Int J Oral Maxillofac Surg 2008;37:625-629. 18499401 10.1016/j.ijom.2008.04.005

[21] Guo C Zhang X Gao F Wang L Sun T: Surgical management of proximal fibular tumors: Risk factors for recurrence and complications. J Int Med Res 2018;46:1884-1892. 29557227 10.1177/0300060518762677 PMC5991257

[22] Sarma A Borgohain B Saikia B: Proximal tibiofibular joint: Rendezvous with a forgotten articulation. Indian J Orthop 2015;49:489-495. 26538753 10.4103/0019-5413.164041 PMC4598538

[23] Sekiya JK Kuhn JE: Instability of the proximal tibiofibular joint. J Am Acad Orthop Surg 2003;11:120-128. 12670138 10.5435/00124635-200303000-00006

[24] Alves-da-Silva T Guerra-Pinto F Matias R Pessoa P: Kinematics of the proximal tibiofibular joint is influenced by ligament integrity, knee and ankle mobility: An exploratory cadaver study. Knee Surg Sports Traumatol Arthrosc 2019;27:405-411. 30056605 10.1007/s00167-018-5070-8

[25] Hakim DN Pelly T Kulendran M Caris JA: Benign tumours of the bone: A review. J Bone Oncol 2015;4:37-41. 26579486 10.1016/j.jbo.2015.02.001 PMC4620948

[26] Kransdorf MJ Stull MA Gilkey FW Moser RP Jr: Osteoid osteoma. Radiographics 1991;11:671-696. 1887121 10.1148/radiographics.11.4.1887121

[27] Marco RA Gitelis S Brebach GT Healey JH: Cartilage tumors: Evaluation and treatment. J Am Acad Orthop Surg 2000;8:292-304. 11029557 10.5435/00124635-200009000-00003

[28] Tsuda Y Gregory JJ Fujiwara T Abudu S: Secondary chondrosarcoma arising from osteochondroma: Outcomes and prognostic factors. Bone Joint J 2019;101-b:1313-1320. 31564158 10.1302/0301-620X.101B9.BJJ-2019-0190.R1

[29] Rapp TB Ward JP Alaia MJ. Aneurysmal bone cyst. J Am Acad Orthop Surg 2012;20:233-241. 22474093 10.5435/JAAOS-20-04-233

[30] Raskin KA Schwab JH Mankin HJ Springfield DS Hornicek FJ: Giant cell tumor of bone. J Am Acad Orthopaedic Surgeons 2013;21:118-126. 10.5435/JAAOS-21-02-118 23378375

[31] Bickels J Wittig JC: Operative Techniques in Orthopaedic Surgical Oncology. Philadelphia, Lippincott Williams & Wilkins, 2012.

[32] Yildiz C Erler K Atesalp AS Basbozkurt M: Benign bone tumors in children. Curr Opin Pediatr 2003;15:58-67. 12544273 10.1097/00008480-200302000-00010

[33] Wyers MR: Evaluation of pediatric bone lesions. Pediatr Radiol 2010;40:468-473. 20225104 10.1007/s00247-010-1547-4

[34] Woertler K: Benign bone tumors and tumor-like lesions: Value of cross-sectional imaging. Eur Radiol 2003;13:1820-1835. 12700923 10.1007/s00330-003-1902-z

[35] Takeda A Tsuchiya H Mori Y Tanaka S Kikuchi S Tomita K: Anatomical aspects of biopsy of the proximal fibula. Int Orthop 2001;24:335-337. 11294425 10.1007/s002640000185 PMC3619925

[36] Arikan Y Misir A Gur V Kizkapan TB Dincel YM Akman YE: Clinical and radiologic outcomes following resection of primary proximal fibula tumors: Proximal fibula resection outcomes. J Orthop Surg 2019;27:2309499019837411. 10.1177/2309499019837411 30909790

[37] Kneisl JS Simon MA: Medical management compared with operative treatment for osteoid-osteoma. J Bone Joint Surg Am 1992;74:179-185. 1541612

[38] Mark Miller ST: Miller's Review of Orthopaedics, ed 7th Edition. Philadelphia, USA: Elsevier, 2015.

[39] Pfeiffer M Sluga M Windhager R Dominkus M Kotz R: Surgical treatment of osteoid osteoma of the extremities. Z Orthop Ihre Grenzgeb 2003;141:345-348. 12822085 10.1055/s-2003-40085

[40] Marquass B Hepp P Theopold JD von Dercks N Blattert TR Josten C: Osteoid osteoma of the proximal fibula: An uncommon location with the indication for open surgery. Case Rep Orthop 2011;2011:324650. 23198208 10.1155/2011/324650 PMC3504221

[41] Malawer MM: Surgical management of aggressive and malignant tumors of the proximal fibula. Clin Orthop Relat Res 1984:172-181. 6723139

[42] Bickels J Kollender Y Pritsch T Meller I Malawer MM: Knee stability after resection of the proximal fibula. Clin Orthop Relat Res 2007;454:198-201. 16936590 10.1097/01.blo.0000238781.19692.16

[43] Pulatkan A Uçan V Tokdemir S Elmalı N Gürkan V: Use of cement combined grafting in upper and lower extremity benign bone tumors. Joint Dis Relat Surg 2020;31:335-340. 10.5606/ehc.2020.71918 PMC7489186 32584734

[44] Zuo D Zheng L Sun W Fu D Hua Y Cai Z: Contemporary adjuvant polymethyl methacrylate cementation optimally limits recurrence in primary giant cell tumor of bone patients compared to bone grafting: A systematic review and meta-analysis. World J Surg Oncol 2013;11:156. 23866921 10.1186/1477-7819-11-156 PMC3717274

[45] Gortzak Y Kandel R Deheshi B: The efficacy of chemical adjuvants on giant-cell tumour of bone. An in vitro study. J Bone Joint Surg Br 2010;92:1475-1479. 21089702 10.1302/0301-620x.92b10.23495

[46] Omlor GW Lange J Streit M: Retrospective analysis of 51 intralesionally treated cases with progressed giant cell tumor of the bone: Local adjuvant use of hydrogen peroxide reduces the risk for tumor recurrence. World J Surg Oncol 2019;17:73. 31014317 10.1186/s12957-019-1613-9 PMC6480805

[47] Meller I Weinbroum A Bickels J: Fifteen years of bone tumor cryosurgery: A single-center experience of 440 procedures and long-term follow-up. Eur J Surg Oncol 2008;34:921-927. 18158228 10.1016/j.ejso.2007.11.001

[48] Futani H Kumanishi S Minakawa GO Yoshiya S: Osteoscopic surgery of giant cell tumor of bone for preservation of proximal fibula. Anticancer Res 2018;38:2995-3000. 29715130 10.21873/anticanres.12552

[49] Campanacci M Baldini N Boriani S Sudanese A: Giant-cell tumor of bone. J Bone Joint Surg Am 1987;69:106-114. 3805057

[50] Humbert ET Mehlman C Crawford AH: Two cases of osteochondroma recurrence after surgical resection. Am J Orthop 2001;30:62-64. 11198832

[51] Alabdullrahman LW Byerly DW: Osteochondroma, in *StatPearls*. Treasure Island, FL, StatPearls Publishing, 2021. 31335016

[52] Dahlin DC Ivins JC: Benign chondroblastoma. A study of 125 cases. Cancer 1972;30:401-413. 5051664 10.1002/1097-0142(197208)30:2<401::aid-cncr2820300216>3.0.co;2-b

[53] Bauer HC Brosjö O Kreicbergs A Lindholm J: Low risk of recurrence of enchondroma and low-grade chondrosarcoma in extremities. 80 patients followed for 2-25 years. Acta Orthop Scand 1995;66:283-288. 7604716 10.3109/17453679508995543

[54] Lam Y Keil L Esther R Kane SF: Bone tumors. FP Essentials, 2020, pp COV+.

[55] Inatani H Yamamoto N Hayashi K: Surgical management of proximal fibular tumors: A report of 12 cases. J Bone Oncol 2016;5:163-166. 28008377 10.1016/j.jbo.2016.06.001 PMC5154704

[56] Faezypour H Davis AM Griffin AM Bell RS: Giant cell tumor of the proximal fibula: Surgical management. J Surg Oncol 1996;61:34-37. 8544457 10.1002/(SICI)1096-9098(199601)61:1<34::AID-JSO8>3.0.CO;2-T

[57] Sakamoto A Okamoto T Matsuda S: A posterior approach for curettage in giant cell tumor of bone in the proximal fibula. J Surg Case Rep 2019;2019:rjz252. 31548873 10.1093/jscr/rjz252 PMC6748765

[58] Dormans JP Hanna BG Johnston DR Khurana JS: Surgical treatment and recurrence rate of aneurysmal bone cysts in children. Clin Orthop Relat Res 2004;421:205-211. 10.1097/01.blo.0000126336.46604.e1 15123949

[59] Rahman MA El Masry AM Azmy SI: Review of 16 cases of aneurysmal bone cyst in the proximal femur treated by extended curettage and cryosurgery with reconstruction using autogenous nonvascularized fibula graft. J Orthop Surg 2018;26:2309499018783905. 10.1177/2309499018783905 29954245

[60] Vergel De Dios AM Bond JR Shives TC McLeod RA Unni KK: Aneurysmal bone cyst. A clinicopathologic study of 238 cases. Cancer 1992;69:2921-2931. 1591685 10.1002/1097-0142(19920615)69:12<2921::aid-cncr2820691210>3.0.co;2-e

[61] Campanacci M Capanna R Picci P: Unicameral and aneurysmal bone cysts. Clin Orthop Relat Res 1986:25-36. 3956013

[62] Lampasi M Magnani M Donzelli O: Aneurysmal bone cysts of the distal fibula in children: Long-term results of curettage and resection in nine patients. J Bone Joint Surg Br 2007;89:1356-1362. 17957078 10.1302/0301-620X.89B10.19375

[63] Mavrogenis AF Rossi G Rimondi E Ruggieri P: Successful treatment of aneurysmal bone cyst of the proximal fibula with embolization. Eur J Orthop Surg Traumatol 2012;22(suppl 1):199-204. 26662777 10.1007/s00590-012-1013-0

[64] Rossi G Rimondi E Bartalena T: Selective arterial embolization of 36 aneurysmal bone cysts of the skeleton with N-2-butyl cyanoacrylate. Skeletal Radiol 2010;39:161-167. 19669138 10.1007/s00256-009-0757-z

[65] Gitelis S Mallin BA Piasecki P Turner F: Intralesional excision compared with en bloc resection for giant-cell tumors of bone. J Bone Joint Surg Am 1993;75:1648-1655. 8245057 10.2106/00004623-199311000-00009

[66] Zhao SC Zhang CQ Zhang CL: Reconstruction of lateral knee joint stability following resection of proximal fibula tumors. Exp Ther Med 2014;7:405-410. 24396415 10.3892/etm.2013.1429 PMC3881052

[67] Agarwal DK Saseendar S Patro DK Menon J: Outcomes and complications of fibular head resection. Strateg Trauma Limb Reconstr 2012;7:27-32. 10.1007/s11751-012-0133-8 PMC3332321 22467142

